# The Successful Doctor

**Published:** 1881-01

**Authors:** T. J. Wentworth


ARTICLE VII.

### THE SUCCESSFUL DOCTOR.

### CLEANLINESS THE KEYSTONE OF SUCCESS.

BY T. J. WENTWORTH.

All that is good is pure, without, within,
A cleanly life 's to godliness akin.
The man of decent habits, clean, refined,
Is one who owns nobility of mind.
Whose soul surmounts the passion's heated sway
And lives, a higher impulse to obey.
Oft have I wished, if 't were my lot to choose,
In minds of men this virtue to infuse.
In souls where Science her fair throne had set,
And sprang the fountain of bright Intellect;
Wherein the tree of Reason throve in growth,
Yet witless wights, these men were slaves of sloth,
Who, chained by Habit's shackles, dared not brave,
But sunk their moral courage in its grave.
Fair law of Nature, let me bow to thee,
What else I am I pray I clean may be,
Let him who will the contrary profess,
'Tis cleanliness the keystone of success.
O ye of dental science, heed I pray

These simple precepts I before you lay.
Let him who would succeed this bear in mind,
He must be willing, quick, affable, kind ;
Love his profession, study well his art,
Do justice to his patients ; have a heart
To feel and sympathize with human pains,
Nor solely bow to paltry, worldly gains.
Let nobler motives instigate his plans
And let his life be a true gentleman's.
Note well his health, herein a secret lies,
And oft from this one source strange ills arise.
This, not for his sole good alone, I tell,
But for his gentle patient's ease as well,
Th' offense is rank indeed when bending o'er
The face of some fair form of beaut'ous pow'r
To belch the rancid breath, with poisonous freight
Of Bacchus' fumes. The nostrils wide dilate,
And from the lungs she strives the flood t' abate
And mentally deplores th' unwholesome fate.
Drive the dyspeptic ill from gastric haunt,
Bid vile tobacco fumes henceforth avaunt,
About the room let everything be neat
Th' instruments, water, spittoon clean and sweet,
Nor with bespattered blood on no pretense
To senses of the gentle give offence.
Have basin, ewer, linen fine and clean,
Wash well the hands, and let the act be seen,
Lest the imagination had conceived
The former patient's ulcerous tooth relieved,
And forthwith from th' obnoxious place decayed,
Th' offending digit to her mouth conveyed.
"The apparel oft proclaims the man," if so
'T were well the nature of the clause to know,
And those same precepts which Polonius gave,
Th' unstudied course of Leartes to save,
May aptly fit the modern man to-day
As he who lived beneath the Danish sway.

Be neat, not gaudy ; rich as purse allows,
Yet let not habit foppish taste arouse,
But store the mind with knowledge, science, truth,
And grasp the mental elixir of youth.
If this be done success will pave its way
As sure as night follows the brightest day.
These precepts may be equally applied
To sons of Esculapius, allied
To those who from the dental school arise
For side by side ofttimes their calling lies.

